# Leveraging Multi-Sectoral Partnership for Colorectal Cancer Education and Screening in the African American Community: A Protocol and Preliminary Results

**DOI:** 10.1007/s13187-024-02506-w

**Published:** 2024-09-23

**Authors:** Jungyoon Kim, Hongying Daisy Dai, Tzeyu Michaud, Sachi Verma, Keyonna M. King, John W. Ewing, Grace Mabiala-Maye, Paul Estabrooks

**Affiliations:** 1 Department of Health Services Research and Administration, College of Public Health, University of Nebraska Medical Center, 984350 Nebraska Medical Center, Omaha, NE 68198 USA; 2 Department of Biostatistics, College of Public Health, University of Nebraska Medical Center, Omaha, NE USA; 3 Department of Health Promotion, College of Public Health, University of Nebraska Medical Center, Omaha, NE USA; 4 Nebraska Hospital Association, Lincoln, NE USA; 5 Douglas County Treasurer Office, Omaha, NE USA; 6 Department of Health and Kinesiology, University of Utah College of Health, Salt Lake City, UT USA

**Keywords:** Cancer education, Multi-sector partnership, Community-based intervention, Fecal immunochemical test, Multi-component approach, Health disparities

## Abstract

Colorectal cancer (CRC) awareness and screening rates are still low in African Americans (AAs), especially for those who do not have regular access to health care. We established a multi-sector community partnership between academia, health system, cancer advocacy, and local county treasurer’s office (CTO), to test a pilot CRC screening intervention using a tailored educational brochure and fecal immunochemical test (FIT). Participants were recruited at a local CTO in an urban midwestern region. Once eligible, participants were assigned to 2-by-2 intervention arms by educational strategy (brochure vs. no brochure) and FIT provision strategy (direct provision by onsite staff vs. indirect provision via phone/online request). We compared the effect of different strategies on FIT return rates. Of 1500 individuals approached, 212 were eligible for the study. The final sample consisted of 209 participants who were predominantly men (57%) and AAs (85%). No differences were found in the return rates by educational brochure (24% [brochure] vs. 23% [no brochure]; *p* = 0.82). In regard to FIT provision strategy, direct FIT provision yielded higher return rates than indirect provision (31% vs. 15%; *p* = 0.01). When the four groups were compared, direct provision with education brochure yielded the highest return rates (33.9%), followed by direct provision only (27.5%), indirect provision only (18%), and indirect provision with a brochure (12.2%). For community-based CRC screening intervention using stool-based test, the direct provision of FIT kits with educational brochure outperforms the other three strategies.

**Supplementary Information:**

The online version contains supplementary material available at 10.1007/s13187-024-02506-w.

## Introduction

Colorectal cancer (CRC) is the third leading cause of new cancer cases and cancer deaths in the United States (US) [1]. CRC can be prevented by undergoing regular screening. The US Preventive Services Taskforce recommended that individuals aged between 45 and 75 years without symptoms or a family history can prevent or detect cancer in earlier stages by undergoing visual examinations (e.g., colonoscopy) or stool-based tests (e.g., fecal occult blood test (FOBT), fecal immunochemical test (FIT), or FIT-DNA test) [2]. Despite the known benefits of CRC screening (CRCS), the current screening rate (71.6%) in the US remains suboptimal. In addition, underserved and minority populations reported lower screening rates compared with the national average. For example, the Behavioral Risk Factor Surveillance System survey data revealed lower rates of up-to-date CRCS for African Americans (AAs) than for Whites (66.4% vs. 70.4%) [3]. The American Cancer Society reported that AAs are 20% more likely to develop CRC and 40% more likely to die from this condition compared with other racial groups [4]. The high CRC incidence and mortality among AAs may be attributed to the limited access to quality healthcare, lower level of cancer knowledge, and timely screening. Studies have indicated that the CRCS rates were notably lower for AAs without a USOC or without insurance (26% and 15%) than for those with a USOC or any form of insurance (65.2% and 68%) [5]. Only 16% of the AA men passed a colon cancer knowledge test, indicating significantly lower levels of colon cancer education and knowledge compared to other racial/ethnic groups [6].

Although colonoscopy is a primary method for CRCS, structural barriers exist, including a lack of access to insurance, transportation-related challenges, difficulty securing childcare services, or insufficient paid time off from work [7]. Recently, stool-based tests (FITs or FOBTs) have demonstrated high acceptability among low-income and minority communities [8]. Numerous randomized controlled trials (RCTs) and systematic reviews have supported the application of stool-based tests in various settings [9, 10]. The current literature highlights the importance of employing multi-component approaches to alleviate structural barriers for AAs, such as the provision of stool-based kits combined with patient navigation, tailored educational materials, and follow-up reminders [10]. In many of these studies, educational interventions (e.g., brochures or pamphlets) [11, 12] and “direct provision” of stool-based tests [13, 14] were the two most notably recognized evidence in improving screening adherence. However, many of these studies were primarily conducted in health system– or health plan–based settings. Therefore, additional evidence is needed in community settings to determine the best practices of these strategies.

The present study described the study design and preliminary results of a pilot, feasibility trial of an innovative community-based CRCS program that was operationalized through a multi-sectoral partnership. The results of this study will serve as a basis for the development of large-scale effectiveness trials in community settings.

## Methods

### Multi-Sectoral Partnership

Between April and July 2021, the research team held multiple meetings with key stakeholders in the community to discuss multi-sectoral partnership ideas to reduce CRC disparities among AAs. Key stakeholders include representatives from a local county treasurer’s office (CTO), a county health department, a cancer advocacy group, and a federally qualified health center (FQHC) serving mostly AA members. During the meetings, we discussed the idea of using one of the CTO branches as a place for recruiting AAs for a CRCS intervention using evidence-based approaches (education with small media and FIT). The selected CTO branch was in an urban area in the midwestern state, where a relatively greater proportion of AA residents reside (> 30% of all residents in the nearest zip code areas) than in other areas in the state.

The community leaders consist of key representatives from the participating organizations in this multi-sector partnership. The role of these leaders is to ensure that each partner organization fulfills its shared responsibilities in the CRCS program, which include (a) providing space and support for participant recruitment and FIT distribution (CTO), (b) sharing an established infrastructure for FIT sample testing and result notification (cancer advocacy group), (c) offering navigation services for positive follow-ups for participants with limited or no access to healthcare (FQHC), and (d) providing FIT kits, data analytics, and funding support (academic team). The two authors (JE and KK) have served as co-chairs of this community-academic partnership, promoting the effort, working with the community to gain support, and gathering feedback and guidance from the community regarding the program’s design, implementation, evaluation, and dissemination.

### Study Objective and Hypotheses

The study objective was to examine the effect of the different approaches (education and invitation strategies) on FIT return rates. We hypothesized that participants receiving a culturally tailored educational brochure will exhibit a higher FIT return rate compared with those who did not receive a brochure. We also hypothesized that the direct provision of FIT kits at the local CTO will result in higher FIT returns, compared with the indirect provision of FIT kits upon request.

### Study Design

This study used the 2-by-2 trial design to assess the effect of four intervention strategies consisting of a combination of an education strategy (brochure vs. no brochure) and invitation strategy (direct vs. indirect provision) on FIT kit return rates. The participants were recruited from one CTO site in the community (convenient sample) and were allocated with equal chance to one of the four study groups: (1) direct provision with an educational brochure, (2) direct provision only, (3) indirect provision with a brochure, or (4) indirect provision only. For this feasibility study, we chose the minimal sample size (*n* = 50 per arm) based on guidelines for behavioral treatment development research [15].

### Participant Enrollment and Follow-up Process

**Fig. 1:**
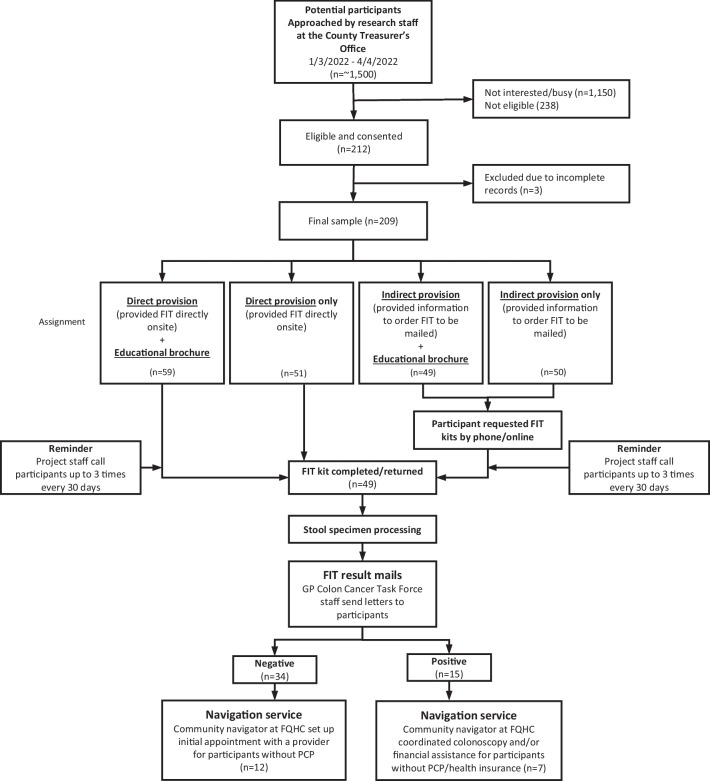
Participant recruitment and program workflow

The eligibility criteria included individuals (1) aged > 19 years given the younger onset of CRC, (2) currently living in the selected county, and (3) who agreed to be contacted for follow-up of test results. As illustrated in Fig. 1, between January 3 and April 4, 2022, our research staff approached adults who visited the CTO site and asked whether they were interested in undergoing a CRCS using a home-based stool test (i.e., FIT). Since the study was conducted during the pandemic, we required all the recruitment staff to follow the public health measures, including COVID-19 vaccinations, wearing masks at the CTO site, maintaining the 6-ft distance, and using hand sanitizers frequently. The impact of COVID on the study was minimal. Those who were deemed eligible were asked to complete an enrollment survey (supporting information file 2). We asked participants for consent to authorize the release of their information for follow-up and to release deidentified information for evaluation purposes. After providing consent, the research staff provided each participant with a packet preassigned to one of the four study arms. A packet for arm 1 (direct provision with a brochure) included an introductory letter (supporting information file 1), a FIT kit, and an educational brochure. A packet for arm 3 (indirect provision with a brochure) included an introductory letter, a brochure, and an informational sheet containing a phone number and an online link through which a FIT kit can be ordered/delivered to their homes. Packets for arms 2 and 4 included the same materials except a brochure. The packets were equally distributed to the participants in the order of arm number. Participants were blinded to the assignment of the intervention. No financial incentives were provided.

Participants received reminder phone calls up to three times approximately every 30 days if they did not return the kit. Of the 209 participants, 14 returned the kit before a call was needed. The remainder (*n* = 195) received follow-up reminders; however, 25 did not have working numbers. Forty-six percent answered the reminder call, and the remaining participants received a reminder via voice mail. FIT samples were returned directly to a designated laboratory, and the test results were mailed within 14–21 days to the participants and their primary care providers (if available) upon agreement. Participants with positive test results and limited healthcare access (i.e., uninsured or without primary doctors) were followed by a health navigator at a local FQHC to schedule them for a colonoscopy and assist them in filing an application for financial support.

### Intervention Strategy

This study used two evidence-based CRCS intervention strategies recommended by the Community Guide [16]. Education using small media (e.g., brochures or postcards) was an effective strategy for increasing CRCS rates. Particularly, tailoring health education messages about CRCS improved the perceptions of message relevance and thus increased the intentions to undergo screening [17]. We developed a 1-page, front-and-back, trifold educational brochure that was culturally tailored for AAs. The contents of the brochure were adapted from the existing resources [18, 19], followed by several rounds of review-and-revise processes with 5–6 key community stakeholders (supporting information file 3). The invitation strategy involved the direct provision of FIT kits (e.g., directly providing the FIT/FOBT kit mostly via mail), which is more effective at promoting CRCS completion than usual care as it eliminates the need to visit a clinic to pick up or request a kit.

### Measures and Data Collection

The primary outcome was FIT return rates at 6 months following the assignment. We obtained participants’ demographic information (age, sex, and median household income based on zip code), CRCS up-to-date status, and health care access (having a USOC/insurance) through the enrollment survey.

### Data Analysis

We used descriptive analyses to summarize the reach and participant characteristics, such as demographic information, health care access, and CRCS up-to-date status. To determine the differences in participant characteristics between the two study arms, chi-square tests and an analysis of variance test were used. In the bivariate analysis, a chi-square test was used to compare the difference in FIT return rates between the two groups by invitation type (direct vs. indirect provision) and educational strategy (education vs. no education) and between the four groups. All analyses were performed using SAS version 9.4 (Cary, NC).

## Results

### Reach and Sample Characteristics

Approximately 1500 individuals were approached by research staff at the CTO. Of them, 212 were eligible. Overall, 209 participants (of 1500 approached; 14% reach) were enrolled in the study. Among them, 59, 51, 49, and 50 participants were assigned to the direct provision with a brochure, direct provision only, indirect provision with a brochure, and indirect provision only groups, respectively. The participants’ mean age was 59 (SD = 12) (Table 1). Most participants were men (56.8%), were non-Hispanic AAs (84.7%), and had a median household income of $21,644. Approximately 38% of the participants either did not have a primary care provider (PCP) or did not provide this information. A quarter of the participants either did not have health insurance or did not provide this information. Most participants did not undergo scheduled CRC screening. No significant differences were found in the participant characteristics among the four study arms.

**Table 1:** Participant characteristics

Variables	Overall	Study arms				*p-*value
		Direct provision + brochure	Direct provision only	Indirect provision + brochure	Indirect provision only	
Sample size	209	59	51	49	50	
Demographic						
Age, mean (SD)	59.0 (11.8)	61.1 (11.9)	58.1 (12.7)	58.4 (9.8)	57.9 (12.4)	0.44
Sex, *n* (%)						
Female	86 (43.2)	22 (37.9)	23 (46.9)	22 (47.8)	19 (41.3)	0.70
Male	113 (56.8)	36 (62.1)	26 (53.1)	24 (52.2)	27 (58.7)	
Race/ethnicity, *n* (%)						
Non-Hispanic Black	172 (84.7)	46 (79.3)	43 (86)	43 (89.6)	40 (85.1)	0.73
Non-Hispanic White	12 (5.9)	5 (8.6)	2 (4.0)	1 (2.1)	4 (8.5)	
Hispanic	8 (3.9)	4 (6.9)	1 (2.0)	2 (4.2)	1 (2.1)	
Others	11 (5.4)	3 (5.2)	4 (8.0)	2 (4.2)	2 (4.3)	
Median household income^a^, mean (SD)	$21,644.0 ($6655.8)	$20,189.1 ($4683.7)	$22,326.5 ($7471.4)	$21,052.9 ($6235.4)	$23,063.5 ($7856.0)	0.21
Healthcare access						
Having a PCP, *n* (%)						
Yes	129 (61.7)	33 (55.9)	35 (68.6)	30 (61.2)	31 (62.0)	0.60
No/not reported	80 (38.3)	26 (44.1)	16 (31.4)	19 (38.8)	19 (38.0)	
Having insurance,* n* (%)						
Yes	157 (75.1)	42 (71.2)	38 (74.5)	37 (75.5)	40 (80.0)	
No/not reported	52 (24.9)	17 (28.8)	13 (25.5)	12 (24.5)	10 (20.0)	0.77
Updated CRC screening, *n* (%)						
Yes	30 (14.3)	8 (13.6)	9 (17.7)	8 (16.3)	5 (10.0)	0.70
No/not reported	179 (85.6)	51 (86.4)	42 (82.4	41 (83.7)	45 (90.0)	

^a^median household income was measured based on ZIP code information collected by the U.S. Census Bureau (2021)

### FIT Kit Return Rates

**Fig. 2:**
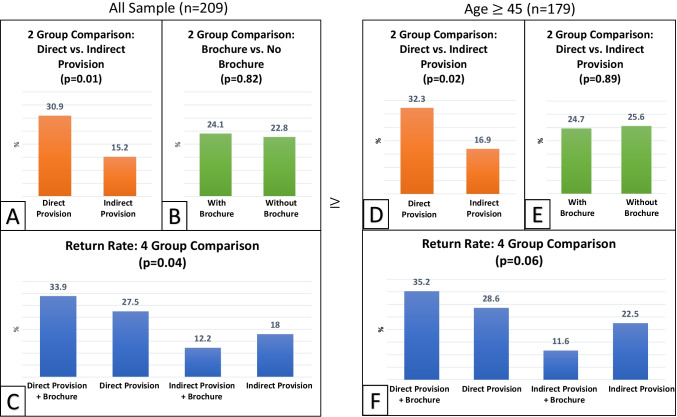
Comparison of the FIT results between the 2 groups and 4 groups

First, we conducted the analysis for all sample (*n* = 209; Fig. 2A–C). In the 2-group comparison (Fig. 2A), the FIT return rate was higher in the direct provision group than in the indirect provision group (30.9% vs. 15.2%, *p* = 0.01). However, no significant difference was observed between the educational brochure group and no-brochure group (24.1% vs. 22.8%, *p* = 0.82; Fig. 2B). In the 4-group comparison (Fig. 2C), significant differences were observed in return rates between the study arms (*p* = 0.04) (direct provision with a brochure: 33.9%, direct provision only: 27.5%, indirect provision with a brochure: 12.2%, and indirect provision only: 18.0%).

We also conducted the analysis for the subsample: individuals 45 years or older (*n* = 179; Fig. 2D–F). Overall, the results were consistent. In the 2-group comparison (Fig. 2D), the FIT return rate was higher in the direct provision group than in the indirect provision group (32.3% vs. 16.9%, *p* = 0.02). There was no significant difference between the brochure group and no-brochure group (24.7% vs. 25.6%, *p* = 0.89; Fig. 2E). In the 4-group comparison (Fig. 2F), a marginally significant difference was observed in return rates between the study arms (*p* = 0.06) (direct provision with a brochure 35.2%, direct provision only 28.6%, indirect provision with a brochure 11.6%, and indirect provision only 22.5%).

## Discussion

The study aimed to increase the CRC awareness and screening among underserved/minority communities through the implementation of a multi-component FIT approach with a multi-sectoral partnership (e.g., CTO). In this study, we identified a CTO, traditionally undervalued for public health promotion, as a community partner for reaching target populations, especially those who have limited access to healthcare. Notably, a greater proportion (~ 25%) of the study sample did not have health insurance or did not report this information, compared with the national uninsured rate for AAs in the US (12%) [20]. Moreover, 40% of the participants either did not have PCPs or did not provide this information, further exacerbating the challenges related to accessing CRCS-related care.

Contrary to our expectations, adding a tailored educational brochure did not increase the FIT return rate. This may infer that providing an educational brochure “alone” would not be sufficient to overcome the access barrier compared with the impact of invitation strategies. Future research is warranted to explore other methods for education, such as a short video or use of audio/visual-aided materials [21, 22]. Recent studies have highlighted the role of smartphone applications or social media campaign for increasing awareness, knowledge, and screening behavior of CRC screening [23, 24]. These complementary education strategies should be determined with the optimal timing and combination of other implementation strategies (e.g., onsite assistance or reminders) [9, 10, 25].

Consistent with the previous literature, providing FIT kits directly at the study site (CTO) was associated with higher return rates than mailing kits upon request [9, 25]. Providing the kit directly eliminates the need to order the kit by phone or online, thus making the entire process more convenient. Our study addresses the knowledge gap by examining FIT invitation strategies (direct vs. indirect provision) in a unique community setting (CTO), where potential participants perform their daily activities without the need to schedule an additional appointment, potentially enhancing accessibility. Our findings further suggest that, for community-based interventions, distributing FIT kits directly at the site (CTO) was more effective than mailing the kits upon request.

This study limitations included a limited generalizability of the study findings due to a convenient sampling approach and a potential self-reporting bias from the enrollment survey measures. As a feasibility study, our eligibility criteria did not fully align with the USPSTF guidelines for individuals aged 45–75 years and who are not up-to-date with CRCS. Our full-scale study will consider these guidelines as our eligibility criterion.

## Conclusion

The study introduced a multi-sectoral partnership to increase CRC awareness and screening in AA community. Our findings showed that the direct provision of FIT kits with educational brochure outperforms the other three strategies in the CTO setting. The study contributes to the existing literature by adding knowledge regarding the best practices of FIT intervention in community-based interventions.

## Supplementary Information

Below is the link to the electronic supplementary material.

Supplementary file1 (PDF 163 KB)

Supplementary file2 (PDF 198 KB)

Supplementary file3 (PDF 700 KB)

## Data Availability

The data presented in this study are available on request from the corresponding author due to data restriction.
