# Supplementary material for: Leveraging Multi-Sectoral Partnership for Colorectal Cancer Education and Screening in the African American Community: A Protocol and Preliminary Results
Source: J Cancer Educ. 2024 Sep 23;40(2):248–55. doi: 10.1007/s13187-024-02506-w (PMC11978712; doi:10.1007/s13187-024-02506-w)
Supplement: Supplementary file 1 — Supplementary file1 (PDF 163 KB) [file 13187_2024_2506_MOESM1_ESM.pdf]

Douglas County Treasurer

John W. Ewing, Jr.
Douglas County Treasurer
15335 West Maple Road, Suite 102
Omaha, NE 68116
Business # (402) 444-7082 Fax # (402) 444-6453
www.dctreasurer.org

November 30, 2021

Dear Community Members,

My name is John W. Ewing, Jr. Many of you might know me, but for those who are not aware, I am the Douglas County Treasurer. You all know that I am a part of our community and only want the best for us.

I would like you to take a moment to think about 'Colon Cancer' today. It is a cancer that occurs in the lower intestine where your food is digested. Sometimes abnormal cells grow, called polyps, which may turn into cancer.

Studies have shown that Black or African American adults are more likely to get colon cancer and die from it, compared to other races/ethnicities. We are collaborating with the Great Plains Colon Cancer Task Force, Charles Drew, and the University of Nebraska Medical Center team to help our community fight this challenge.

However, this cancer is preventable if people get tested early. Stool-based tests are easy and can be done from the comfort of your home.

Please complete the enclosed stool test. It takes only a few minutes. I have done it myself.
If you have any questions, please contact Ms. Emily Sarcone, the program coordinator at the Great Plains Colon Cancer Task Force, at (402) 398-5663. If we do not hear from you, we may contact you by phone or text to remind you.

Sincerely,

John W. Ewing, Jr.
Douglas County Treasurer

Douglas County Treasurer

John W. Ewing, Jr.
Douglas County Treasurer
15335 West Maple Road, Suite 102
Omaha, NE 68116
Business # (402) 444-7082 Fax # (402) 444-6453
www.dctreasurer.org

November 30, 2021

Dear Community Members,

My name is John W. Ewing, Jr. Many of you might know me, but for those who are not aware, I am the Douglas County Treasurer. You all know that I am a part of our community and only want the best for us.

I would like you to take a moment to think about 'Colon Cancer' today. It is a cancer that occurs in the lower intestine where your food is digested. Sometimes abnormal cells grow, called polyps, which may turn into cancer.

Studies have shown that Black or African American adults are more likely to get colon cancer and die from it, compared to other races/ethnicities. We are collaborating with the Great Plains Colon Cancer Task Force, Charles Drew, and the University of Nebraska Medical Center team to help our community fight this challenge.

However, this cancer is preventable if people get tested early. Stool-based tests are easy and can be done from the comfort of your home.

Please use the QR code, or visit https://redcap.link/getscreened if you want us to mail **a free test** for you. You could also contact the program coordinator, Ms. Emily Sarcone at (402) 398-5663. If we do not hear from you, we may contact you by phone or text to remind you.

Sincerely,

John W. Ewing, Jr.
Douglas County Treasurer
