# Supplementary material for: Leveraging Multi-Sectoral Partnership for Colorectal Cancer Education and Screening in the African American Community: A Protocol and Preliminary Results
Source: J Cancer Educ. 2024 Sep 23;40(2):248–55. doi: 10.1007/s13187-024-02506-w (PMC11978712; doi:10.1007/s13187-024-02506-w)
Supplement: Supplementary file 2 — Supplementary file2 (PDF 198 KB) [file 13187_2024_2506_MOESM2_ESM.pdf]

Community Colon Cancer Screening Form
For Men and Women aged 45-75

Distribution Location:
Douglas County Treasurer's Office
(North Omaha location)

GREATPLAINS COLON CANCER TASKFORCE

==

1. **Please answer all questions below.** We will use your information to follow-up your results.
2. Please **read and sign**.
3. Give **COMPLETED** form to the staff. Our staff will hand out a screening packet for you.

First Name		Middle Initial	Last Name	
Birth Date / /	Gender M / F	Address		
City		State		Zip
Phone: ( )			Email:	

What is your race?
☐ Asian
☐ Black/African American
☐ Caucasian/White
☐ Native American/Alaska Native
☐ Two or more races
☐ Other _______________

Are you of Hispanic/Latino origin?
☐ Yes
☐ No
☐ Unknown

If you have a primary care (family) doctor, what is your doctor's name or clinic name?

____

City location of your doctor or clinic: _________________________________

Do you have a health insurance? ☐ Yes (_____________________________) ☐ No

Have you done colorectal cancer screening in the past?
☐ Yes ☐ No
If yes, when was the last time you did colorectal cancer screening?
Date: ___ / ___ / ___ (☐ colonoscopy; ☐ FIT/FOBT; ☐ FIT-DNA; ☐ Other)

Disclosure Statement – This test is used only to find out if there is hidden blood in your stool. Blood in your stool can be a sign of several conditions and does not necessarily mean that you have cancer. A positive test result means that you need a follow-up examination. We will inform your family (primary care) doctor about your test results, so that you can schedule a follow-up examination. If you do not have a physician, a Health Navigator at Charles Drew Health Center, Inc. will reach out to you to help setup a follow-up visit at one of their locations. You may also contact Carliss Miller, Lead Health Navigator at 402-810-9788. A negative result means that you do not have any sign of blood in your stool. We highly recommend you taking this test every year.

Authorization to Release Information – I hereby authorize the release of my stool test results, the information contained on my registration form, and recommended related tests to the testing facility and my doctor. This information, as well as patient and physician identify, will be kept strictly confidential and used only for follow up and evaluation purposes by the Great Plains Colon Cancer Task Force. The recipient of the patient information is prohibited from disclosing the information to any other party and is required to destroy the information after the need has been fulfilled.

Your signature: ____ **Date:** _____ / _____ / ________
