# Supplementary material for: Leveraging Multi-Sectoral Partnership for Colorectal Cancer Education and Screening in the African American Community: A Protocol and Preliminary Results
Source: J Cancer Educ. 2024 Sep 23;40(2):248–55. doi: 10.1007/s13187-024-02506-w (PMC11978712; doi:10.1007/s13187-024-02506-w)
Supplement: Supplementary file 3 — Supplementary file3 (PDF 700 KB) [file 13187_2024_2506_MOESM3_ESM.pdf]

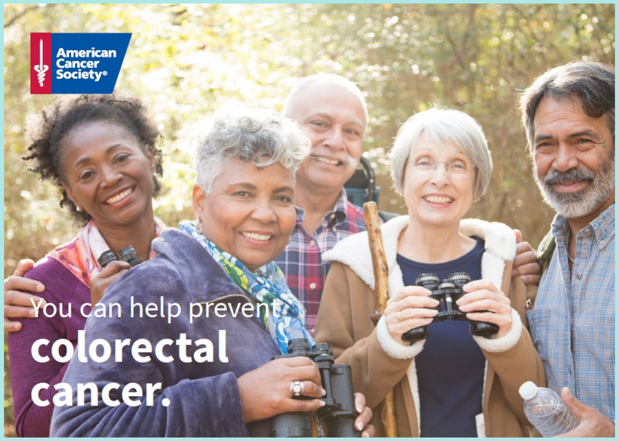

What is colorectal cancer?

It is cancer of the colon or rectum. Most colorectal cancers start with a ***polyp***, a small abnormal growth on the lining of the colon or rectum. Anyone can get colorectal cancer, but it occurs more often in people 45 and older.

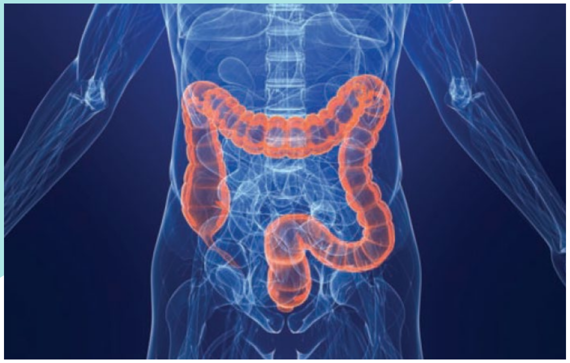

View of the colon and rectum in the human body

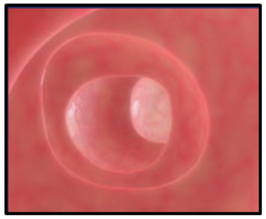

View inside a healthy colon

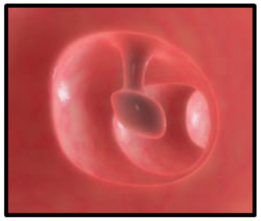

View inside a colon with a polyp

How many people get colorectal cancer?

- About ***1 in 20*** people over age 50 will develop colorectal cancer. It is the 3rd most common type of cancer.
- In the United States, colorectal cancer kills ***1 person every 10 minutes***. It is the 2nd leading cause of cancer death.

Why is it important for Black or African American adults to be aware of colorectal cancer?

- Black or African American adults are about ***20% more likely*** to get colorectal cancer as compared to other racial groups.
- Colorectal cancer is the ***3rd most common*** cancer among Black or African Americans.
- Black or African American adults with colorectal cancer are ***more likely to die*** from it than other ethnic/racial groups.

African American males	African American females
**1 in 23** develop colon or rectum cancer	**1 in 24** develop colon or rectum cancer
**1 in 46** die of colon or rectum cancer	**1 in 51** die of colon or rectum cancer

What are symptoms?

- Blood in the stool or in the toilet after you have a bowel movement
- Diarrhea or constipation over a long period
- Pain or cramping in the abdomen
- Changes in the shape or size of your stool
- Sudden or unexplained weight loss

Talk to your doctor if you have any of these symptoms. They may be caused by something other than cancer. The only way to know for sure is to see your doctor.

I do not have any symptoms. Should I still be screened?

Yes! People with polyps or cancer in the colon or rectum usually do not have symptoms. Colorectal cancer can be found early with a screening test. When found early, the chance of being cured is about ***92 percent.***

How can I protect myself?

If you are 45 and older, ***get screened!***
Look at the back page to see different options for screening.

Find more information at
www.coloncancertaskforce.org

Great Plains Colon Cancer Task Force
7914 W Dodge Road, #348
Omaha, NE 68114
402-295-5071
contact@coloncancertaskforce.org

1-Step vs. 2-Step Colorectal Screening

1-Step Test

1-STEP TEST Colonoscopy is a one-step test that looks for growths called polyps in your entire colon (large intestine) and rectum using a colonoscope. Your doctor can **remove polyps** during colonoscopy and **prevent colorectal cancer**.

COLONOSCOPY
Your doctor can see and remove pre-cancers called polyps and prevent or detect or confirm colorectal cancer
ALL IN 1 STEP.

2-Step Test

2-STEP TESTS If tests such as Fecal Immunochemical Tests (FIT) or multitarget stool DNA **are positive**, a follow up colonoscopy would be required **as a second test**.

1st STEP
Stool-based Test
FIT Test (Fecal Immunochemical Test)
Multitarget Stool SNA

OR

Flexible Sigmoidoscopy

OR

Imaging Test
CT Colonoscopy
Colon Capsule

POSITIVE TEST?

2ND STEP
Colonoscopy

Stool Based Test (FIT)

- A screening test you do at **home**.
- Recommended **once per year**.
- Polyps and cancers may bleed slightly into the stool (feces), but you usually can't see the blood. The **FIT looks for hidden blood in the stool**.

How to prepare for and take the test

- **No special diet** is needed.
- Only one stool sample is needed
- It is important to mail it as soon as you complete the test (**within 3 days**).
- The FIT test kit includes a **pre-paid postage stamped** return envelope (addressed to the lab).

Advantages of this test (the pros)

- You can do the FIT in the privacy of your own **home**.
- You don't need to clean out your colon before the test.
- There are no complications.
- Having this test once per year **lowers your chances** of getting colorectal cancer or dying from it by about **30%**.

Disadvantages of this test (the cons)

- If the FIT is positive (meaning there was blood in your stool), more tests will be needed. A colonoscopy is usually recommended.
- Many people with a positive FIT don't have polyps or cancer (false-positives)
- Some people with a negative FIT do have polyps or cancer (false-negatives).

Visual Examination (Colonoscopy)

- An exam of the **inside** of the **rectum and entire colon**.
- Recommended **once every 10 years**.
- This test looks for polyps or cancer throughout the entire colon.

How to prepare for and take the test

- Your colon needs to be clear for the test (**no solid food the day before the test**).
- Medications can be used to clear stool from your colon.
- To make the test more comfortable, you are given medication to make you sleepy (sedated).
- The exam is done with a lighted tube about the width of a finger. The tube is inserted into the colon through the rectum.

Advantages of this test (the pros)

- This test lets the doctor see the rectum and the entire colon.
- There is **little to no discomfort** while you are sedated.
- Polyps and tissue samples can be taken out during the test.
- Having this test once every 10 years may **lower your chance** of getting colorectal cancer or dying from it by **50% or more**.

Disadvantages of this test (the cons)

- You'll **need a ride home** after the test since you may still be sleepy.
- There's a small chance that the medication will cause a bad reaction.
- There may be some discomfort after the test.
- This test is usually safe, but there is a slight change of complications (e.g., bleeding, perforation, etc.)
